# Preface to *The Journal of Dermatology* special issue: What's new in autoimmune bullous diseases

**DOI:** 10.1111/1346-8138.16683

**Published:** 2023-01-18

**Authors:** Daisuke Tsuruta

**Affiliations:** ^1^ Department of Dermatology Osaka Metropolitan University Graduate School of Medicine Osaka Japan

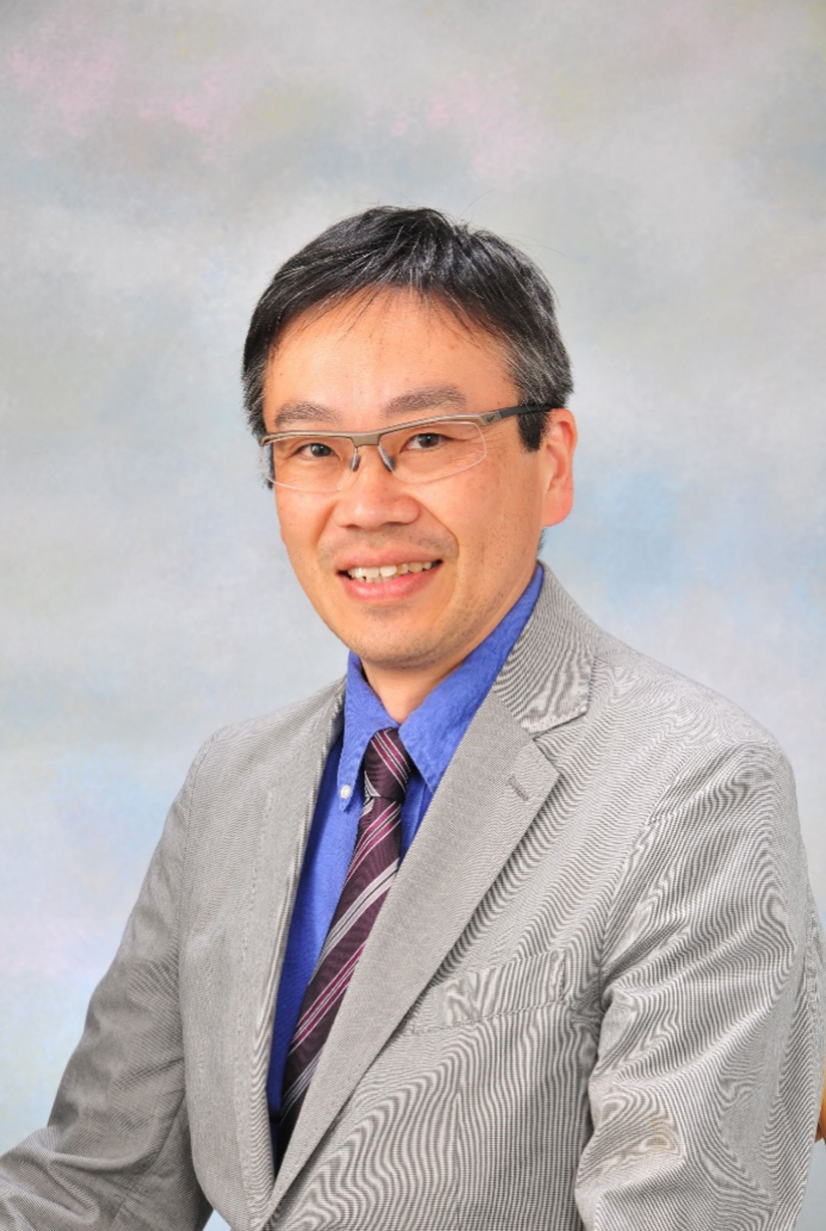

Pemphigus and bullous pemphigoid (BP) are autoimmune bullous diseases that predominantly affect middle‐aged and elderly individuals. The gold standard of treatment is oral corticosteroids. Intravenous immunoglobulin (IVIG), plasmapheresis, and various immunosuppressive agents are also used to treat BP. The use of these drugs in older patients is extremely risky in terms of prognosis, because the side effects of them are so severe. Therefore, we, researchers and physician scientists, should aim to elucidate the pathogenesis of pemphigus and BP, and to establish new, safe treatments with fewer adverse effects. In this special issue, we invited leading Japanese pemphigus and BP physician scientists as authors to update us on how far the pathogenesis of pemphigus and BP has been elucidated and what kind of research is being conducted with the aim of establishing new treatment strategies.

First, three authors submitted state‐of‐the art reviews about pemphigus. Dr Hayato Takahashi described in detail the T cell–mediated immune breakdown against desmoglein 3 in the elimination as “T‐cell autoimmunity and immune regulation to desmoglein 3, a pemphigus autoantigen.” Furthermore, Dr Jun Yamagami summarized the current status of B cell–targeted therapy, namely, rituximab and Bruton tyrosine kinase inhibitor therapy in “B cell–targeted therapy of pemphigus.” In addition, Dr Norito Ishii summarized in detail the role of anti‐desmocollin antibodies, which are found in atypical pemphigus as “Significance of anti‐desmocollin autoantibodies in pemphigus.”

Next, two authors offered reviews on pemphigoid. First, Dr Hideyuki Ujiie described the pathomechanism and trigger of BP in “What's new in the pathogeneses and triggering factors of bullous pemphigoid.” Then, Dr Sho Hiroyasu and our group described the devastating symptom in BP, itch, in “Pruritogens in pemphigoid diseases: possible therapeutic targets for a burdensome symptom.” Furthermore, Dr Yumi Aoyama reported a rare case of bullous pemphigoid.

We believe that by understanding all of these reviews and applying them to research and treatment, we will be able to provide state‐of‐the‐art fields in these diseases at a glance. It will be exciting to see what will be elucidated in this rapidly advancing field of research when a similar special issue is published again in a few years. In particular, I hope that we will be able to understand why these diseases occur by elucidating the initial triggering factor within a few years. It is also clear that progress in research on these two diseases will lead to the elucidation of the pathogenesis of other general autoimmune diseases and guide the development of treatments.

